# Communicating about eating behaviors. A qualitative study of Chilean women and their health-care providers

**DOI:** 10.3402/qhw.v10.25979

**Published:** 2015-02-05

**Authors:** Patricia Gálvez, Alejandra Valencia, Ana M. Palomino, Marjorie Cataldo, Andiara Schwingel

**Affiliations:** 1 Department of Kinesiology and Community Health, University of Illinois at Urbana Champaign, Champaign, IL, USA; 2 Nutrition and Dietetic School, University of Chile, Santiago, Chile

**Keywords:** Health care providers, eating behaviors, woman, eating behavior determinants, public health center

## Abstract

Good communication between health care providers (HCPs) and patients is critical in achieving positive health outcomes. The purpose of this article was to compare the perceptions of Chilean woman and their HCPs with respect to determinants of eating behaviors. Semi-structured interviews were conducted with women (*n*=15) visiting a public health care center in Chile and with their HCPs (*n*=8) who were in charge of promoting healthy eating behaviors among women. Data from the interviews indicated similarities and inconsistencies in determinants of eating behaviors between the groups. Both mentioned many important factors that influence women's eating behaviors, including food preferences, dietary knowledge, self-control and self-efficacy, family, food cost, and food availability. HCPs appeared to be less aware of the role that personality traits and past experiences play as potential determinants which women mentioned. In contrast, women were less aware of the influence of anxiety and low self-esteem on eating choices, which HCPs noted as key factors. Although it was encouraging to see agreement between women and their HCPs in some areas, it is important to work on increasing understanding among the groups with respect to the important role psychological factors play in influencing eating behavior. We suggest that HCPs should focus on the importance of women's personality traits and past eating behaviors, as well as work on improving women's self-esteem and helping to decrease their anxiety levels. HCPs should be encouraged to develop good communication with each person in order to help them understand the roles that external and internal factors play in eating behaviors.

Health care providers (HCPs) protect and care for the population's health. They shoulder the burdens of health promotion, disease prevention, and caring for the sick. As health problems have changed over the decades, new challenges have emerged for HCPs. Currently, the prevalence of non-communicable diseases (NCDs) is greater than that of acute or infectious diseases. This requires HCPs to develop skills in people interaction (World Health Organization, 2005), especially in the area of communication, an essential element in the prevention and treatment of NCDs (Fernstrom, Reed, Rahavi, & Dooher, 2012; Zhao, Villagran, Kreps, & McHorney, 2012). Furthermore, contemporary models of health care rely increasingly on person-centered communication. This person-centered approach asks HCPs to explore people's reasons for visiting them, to understand people's medical and emotional needs, and to strengthen their relationship with the people under their care (Wanzer, Booth-Butterfield, & Gruber, 2004). Such an approach requires that HCPs consider people's needs, worries, perceptions, and opinions in addition to the illness itself. It is important for HCPs to be aware of how each person perceives different illnesses and to take these factors into consideration when developing treatment plans (Ishikawa, Hashimoto, & Kiuchi, 2013). Similarly, each person should be an active participant in their own treatment plan and must understand and agree with any treatment plan proposed by their HCPs (Ishikawa et al., 2013; Wanzer et al., 2004). In order for this to happen, it is necessary that the person feel comfortable expressing what he or she is experiencing to the HCPs, requiring good communication skills from both parties (Alexander, Hearld, Mittler, & Harvey, 2012; Cegala, Street, & Clinch, 2007; Harrington, 2004). Bergsten, Bergman, Fridlund, and Arvidsson (2011) indicate that person-provider communication is essential “to create shared meaning through good dialogue between the parts” (p. 2).

In Chile, responsibility for the promotion of healthy lifestyles and prevention of diseases falls primarily on the primary health care system, following the principles articulated in the Declaration of Alma-Ata (World Health Organization, 1978). The primary health care system is composed of institutions called “health care centers” in which a designated number of community members are served by a multidisciplinary team of HCPs (Alarcón, Torres, & Barna, 2012). Led by these HCPs, Chilean health centers offer a variety of health promotion programs. One of the main health concerns addressed in these programs is the widespread consumption of unhealthy diets, considered to be one of the most important modifiable risk factors underlying the global prevalence of NCDs (Nishida, Uauy, Kumanyika, & Shetty, 2004; World Health Organization, 2014).

Unhealthy diets include foods with high amounts of fat, salt, and sugars; and low amounts of vitamins and minerals (World Health Organization, 2014). The Chilean population intakes, on average, 9.8 g of salt per day (96% over the recommended daily allowance) and only 15% of the total population (18% of women and 13% of men) consume the daily recommended five servings of fruit and vegetables (Ministerio de Salud de Chile, 2010). Despite efforts from the Ministry of Health to improve nutrition habits in Chile, the health consequences of an unhealthy diet have contributed to the prevalence of obesity and diabetes. For example, 39.3% of the population over 15 years old is now considered overweight, and 25% are considered obese. Women are statistically more likely to be obese than men, at 30.7 vs. 19.2%, respectively, even as rates of obesity continue to increase throughout the country (Ministerio de Salud de Chile, 2010). A possible explanation for the poor outcomes of health promotion programs to date could be the poor communication between people and their HCPs, leading to confusion and misunderstandings related to treatment plans. A number of factors need to be taken into consideration when designing effective health promotion programs, including an appreciation for the limited time HCPs can spend with each person, the importance of considering psychological aspects of behavior change, and the critical need for effective communication to avoid misunderstandings (Holtrop, Dosh, Torres, & Thum, 2008).

This study is focused on exploring the degree of agreement between HCPs and women they care for in the public health care system with respect to important health behaviors surrounding healthy eating. The goal was to evaluate the degree of shared understanding regarding the causes of poor eating behaviors in a public health care center in Santiago, Chile. Few studies have explored this particular topic; furthermore, there are even fewer studies that consider communication between people and HCPs other than physicians. This issue has not been studied directly in Chile, and there are few studies that have explored issues related to the determinants of eating behaviors using qualitative research methodology (Olivares, Bustos, Moreno, Lera, & Cortez, 2006; Troncoso & Amaya, 2009).

Qualitative research is considered useful for understanding the determinants that underlie complex human behaviors from a perspective that is quite different from what can be achieved using more traditional quantitative experimental designs. According to Bisogni, Jastran, Seligson, and Thompson (2012), qualitative methods have the potential to better illuminate the “social and behavioral aspects” of eating behaviors (p. 282). Qualitative methodologies have been valued to study eating behaviors (Swift & Tischler, 2010). This study's purpose is to contribute to the understanding of the effects of the perceptions of Chilean women and their HCPs and to discern how they critically influence important determinants of eating behavior.

## Methods

### Participants and site

Study participants consisted of two groups. Group 1 included 17 Chilean women aged between 31 and 58 years, recruited from a Chilean public health care center in Santiago, Chile. Recruitment occurred primarily in the waiting room of the Health Center. Twelve women were of low socioeconomic status, defined as having a monthly income of less than $300 per capita (Instituto Nacional de Estadistica, 2013). Thirty-five percent of participants presented with at least one NCD, 55% self-identified as housewives, 13% were overweight, and 40% were obese (BMI ≥25 kg/m^2^). Table I shows general characteristics of the women. Two women did not complete the data collection, so our final sample included 15 participants. Group 2 included eight HCPs (seven women) who worked at the same Chilean public health care center; among them were four dietitians, three obstetricians, and one nurse. The HCPs were responsible for promoting healthy eating for the women in Group 1. A convenience sample of volunteers was used for both groups.

**Table I:** General characteristics of women (Group 1).

Variable	Label	*n*	%
Marital status	Single	4	26.7
	Married	9	60.0
	Divorced	1	6.7
	Living with the partner	1	6.7
Having children	Yes	13	86.7
Number of children, range	2–6	—	—
Age of children, range	2–37	—	—
Highest educational level	Less than 12th grade	1	6.7
	High school	7	46.7
	Technical	5	33.3
	College	1	6.7
	Master degree	1	6.7
Kind of health insurance	Public	14	93.3
	Private	1	6.7
Work status	Unemployed	6	40
	Employed	9	60

### Data collection

Semi-structured interviews were conducted in Spanish by a trained researcher. Because of the behavioral basis of this study, the interview protocol included questions covering topics related to the constructs of health behavior theories. These theories included the socio-ecological model (Dresler-Hawke & Veer, 2006), social cognitive theory (Anderson, Winett, & Wojcik, 2007), the theory of planned behavior (Bhuyan, 2011), and the health belief model (Orji & Mandryk, 2014). These theories were included as several studies had used them in relation to understanding eating behaviors (Anderson et al., 2007; Kothe, Mullan, & Butow, 2012; Lindsay, Sussner, Greaney, & Peterson, 2009). In addition, these theories can help explain the causes and consequences of complex eating behaviors (Shumaker, Ockene, & Riekert, 2009). Questions were designed for both groups based on constructs from the study goals and behavior theories. The goal of these questions was to obtain the perceptions (of women and HCPs) about what factors were influencing the women's eating behavior. Table II includes an illustrative sample of questions to show the way in which interview questions were posed to both groups.

**Table II:** Examples of questions from protocol interview.

Questions for Group 1
Are there people who are influencing (positively or negatively) your eating behavior? Who are they? What do you think about your community and its access to food in general? In your opinion, what is the relationship between the diet a person has and his or her health? Have you ever intended to change your diet? Why?
Questions for Group 2
What are the factors that are influencing the women's diet? Do you think that there are internal factors such as beliefs, knowledge, or others that are influencing the women's diet? Why? How is the environment influencing the women's diet? Why?

Group 1 included women; Group 2 included health care providers. These questions are only examples of the interview guide.

In Group 1, interviews were performed using photo-elicitation technique; that is including pictures during an interview (Harper, 2002). Past studies using photo-elicitation with vulnerable populations report that the method is useful in capturing participants’ perceptions, experiences, and beliefs especially among participants with lower educational attainment (Baker & Wang, 2006; Yamasaki, 2010). At the time of recruitment, each woman received a disposable camera, and was asked to take approximately 25 pictures of their food and nutrition world. During the personal interviews, the pictures were used to allow women to express their ideas more broadly and to stimulate their memories, in order to obtain detailed information about the determinants of their eating behavior. Interview questions were asked in a way that allowed participants to take advantage of the information depicted in their photographs.

The interviews with the women were conducted in convenient locations, such as in the health care center offices, or places located directly in front of the health center. One interview was done at a participant's home. The average interview lasted from 40 to 60 min. Prior to the interview, information concerning eating habits was collected using a 24-h recall questionnaire for 3 non-consecutive days (Ma et al., 2009). Interviews with HCPs (Group 2) were conducted in their offices and lasted from 25 to 75 min. The time for the interviews with HCPs was affected by their agenda and their availability. Interviews with HCPs did not use photo-elicitation and were focused on their perceptions about the women under their care.

Prior to recruitment and data collection, authorizations from the Institutional Review Board at the University of Illinois at Urbana-Champaign and from the Health Care Center's Ethics Committee in Chile were obtained (June to August 2013). Informed consent was obtained from all participants prior to their initiation in the study.

### Data analysis

All interviews were transcribed, checked for accuracy, and analyzed using thematic analysis (Braun & Clarke, 2006) by three Spanish-speaking investigators (Chilean nutritionists). Initially, each researcher separately coded and analyzed the data for themes using Nvivo10 Software (QSR International, 2014). Each created codes that focused on implicit (what was understood by the research from what the women and HCPs mentioned) and explicit (what was mentioned directly by the women and HCPs) concepts or ideas about factors determining eating behavior. In the first stage of the data analysis, we used inductive analysis (without preconceptions about the issue). After that, the researchers guided a second data revision focused on the research question (deductive analysis) and theories of health behaviors. Subsequently, the team compared codes for agreement, retaining only those themes that were coded by the majority of investigators and unanimously agreed upon by the entire team after extensive discussion. The final themes represent the perspectives of the majority of participants. When negative cases arose, the team discussed each case, using the discussions as an opportunity to further refine each theme (Patton, 2002). To analyze the degree of communication between both groups, after this final revision, the research team completed a comparative analysis in which codes found in interviews from the women were compared to those from the HCPs. This comparison allowed the team to look for agreements and disagreements between the two groups. The final codes were grouped in themes; quotes from individual interviews are provided as examples of the themes identified.

## Results

**Figure 1:**
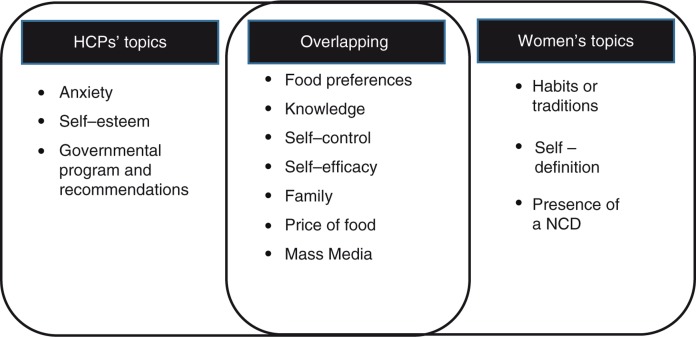
Schema of themes found in health care providers and their women patients. NCD, non-communicable disease.

The themes that emerged from the transcripts were organized into two overarching categories: 1) themes that emerged from both HCPs and women and 2) unique themes that emerged from only one of the groups. A schematic representation of the findings is presented in Figure 1.

### Themes that emerged from women and HCPs

#### Preferences are the most important

Women identified personal food preferences as one of the main determinants of their eating behavior. Women expressed that they eat certain kinds of food because they liked them and refrained from those they did not. Examples of women's quotes are as follows:

I do not like it [cheese], or milk … I like sweet milk …, (WEF)

I think that this [coffee] is delicious with this milk … I like it …, (WMM)

… because they are delicious [fries], they like us …, (WAC)

… because I do not like the flavor [a soft drink] …. (WMP)

HCPs also recognized this variable as a determinant of eating behavior, which they expressed with comments such as:

Simply because they [women] like them. (HCPCG)

#### Women know

Knowledge was another important factor that emerged in food selection and intake—especially knowledge and understanding of healthy eating. Many women demonstrated that they are knowledgeable about food, specifically about the relationship between food and health. Most of the women chose specific foods because they knew about some health-related characteristic of the food. For instance one woman said,

I understand that you have to eat vegetables to have good digestion. (WMR)

Another woman stated that she eats legumes because they are good for her digestion. (WEF)

Because [HCPs] say that we have to eat less sugar, sugar is bad, and instead of this we use sweetener. (WLB)

Overall, women often associated their food choices with health. This point was illustrated by a woman who said:

Depending on the way that you eat and the things that you eat, you are going to be healthy. (WMM)

For the HCPs, knowledge was considered to be a critical factor that could help women follow their dietary recommendations. They recognized that women have some knowledge about food, but probably not enough. For example, some HCPs thought that women generally do not know enough about different varieties of food or methods of cooking. One HCP said,

People do not know many vegetables; in reality they only stick with lettuce …. (HCPD)

Additionally, some HCPs stated that there is a difference between what women know and what they actually eat. This may be true because, in our study, some women reported that they only eat easily available foods, depending on the cost of food and an availability of time to cook.

#### A weakness: lack of self-control and self-efficacy

Self-control was mentioned by both groups who reported that many women lack the capability to control specific eating behaviors. For the women, a sense of lacking control was revealed in some quotes such as:

I see a chocolate bar and if I continue to see it, I am going to eat the entire bar. (WEF)

If I am tempted with fries … I will eat them, (WJR)

… because I am tempted to eat. (WCV)

Because of their reported lack of self-control, HCPs thought that the women needed some external assistance to maintain a healthy diet, such as direct supervision from HCPs.

Both groups agreed that women often lack the self-efficacy to eat well. Self-efficacy is defined as the “confidence in one's ability to take action” (Glanz, Rimer, & Viswanath, 2008, p. 48).The women were asked to evaluate how capable they felt of improving the way that they eat, on a scale from 1 to 10 (where 1 means not at all, and 10 means a great deal); only one woman answered with a 10. The rest of the women indicated that they were between a 5 or 6. When women were asked about why they evaluated themselves at such a low point in the scale, they commented as follows:

… It is difficult for me to follow a habit …, (WCV),

I could do it … but I cannot do it because I am tempted …. (WJR)

According to HCPs, this lack of self-efficacy was thought to be related to willpower or motivation rather than a lack of knowledge. Low willpower could be an indirect variable that influences eating behaviors mentioned by HCPs (Barberia, Attree, & Todd, 2008).

#### Strong family influence

Family was an important external determinant of eating behaviors for many women. Many women did not recognize their family's influence on their behaviors when they were asked directly about it. However, the women mentioned their families often during the interviews, especially their partners:

My husband is used to having in his house [in reference to her husband parents’ house], many different things, so he gets sad if I only have bread and butter; for me this does not matter, but for him …. (WMR)

… in my house, my husband likes of fat food such as “pernil” [a Chilean food based on fat and pork] … he sometimes brings it to home and say “okay, let's go to eat the pernil in the tea time” … sometimes my husband makes us [she and her kids] fall into temptation. (WAC)

He loves chocolate, so we go to the supermarket and he says “buy two chocolates.” To take two chocolate means that at night he goes to the bed, and takes the chocolate to the bedroom, divides it and leaves on the bed …. (WJR)

Women also mentioned that their partners bring home chocolates, fatty foods, and others unhealthy food to demonstrate their love. Women also indicated that they changed their habits after getting married.

The HCPs also identified partners as one of the most important determinants influencing woman's food choices, purchases, and food intake. For example, an HCP said

If the women go buy food with their husbands, it is probable that the cart includes things the women do not want to buy …. (HCPJC)

HCPs also mentioned that children can have negative influences, and reported a belief that women are more concerned with their children eating well and were often not concerned with their own eating practices. One provider said that a typical comment she had heard was that women have to do a lot of things for their children, so they often have no time to eat well.

She wants the best for her children not for herself because … she feels that she does not deserve it or does not have the time or the mother does not think of herself …. (HCPD)

Also, a woman mentioned that if there was a shortage of food at home due to a lack of resources, she preferred to give the scarce resources to the children. Another woman said:

If I see that there remain only three pieces of cheese, I leave them to my husband and my kids. (WPM)

#### Cost matters

The price of food was also mentioned by both groups. This factor greatly influences several aspects related to food and eating: the place where women buy food, what they select, availability of food at home, what they cook, and what they eat. For example, most women indicated that they buy fruits and vegetables at the farmers market because the food is cheaper. Other kinds of food—such as rice, pasta, and meat—the women bought in supermarkets for the same reason. Also, women indicated that when a food item is more expensive, they typically do not buy it: “when [a type of] food is so expensive I do not eat them and nobody eats it” (WPV). According to the HCPs, women eat fast food or rice, pasta, and other cereals because they are cheaper than vegetables, and provide enough food for the entire family. They recognized that sometimes the women's economic situation made it difficult for them to follow a specialized diet, although they did acknowledge that women have the perception that a healthy diet is always more expensive.

#### Media influences

Mass media, especially television, was thought to influence eating behaviors because they act as sources of knowledge and understanding (or misunderstanding). Women indicated that they do watch TV programs where nutritionists and nurses talk about diets. They also mentioned hearing some governmental recommendations through the media, mainly those related to increasing the intake of fruit and vegetables. The HCPs said several TV programs promote new products and they sometimes feel that they are competing with them because famous people promote foods that are not always consistent with a healthy diet.

### Unique themes that emerged from only one of the groups

#### Women: my old (bad) habits

A topic mentioned only by the women, and not the HCPs, was old habits or traditions. These habits frequently come from their upbringing or habitual behaviors. Women described these types of behaviors as difficult to change. For example, a woman said

Always there is salad in my house … It is a tradition from my parents. (WLB)

In my house, they [the woman's parents] always have been carnivores … they do not eat meals without meat, and I also do this. (WJR)

#### Women: guided by my self-definition

Women identified themselves with certain personality characteristics that led them to eat more of a specific type of food. Two women mentioned that they were greedy and for this reason they eat cakes. One women stated: “I am lazy” (WPM) to indicate that she could not organize her life to change her bad eating behaviors. Other women defined themselves as “tempted” and for this reason they cannot abstain from certain kinds of food. Another woman defined herself in relation with her family

I am grandmother, so I have to take care of myself [and eat healthy] due to my grand kids …. (WJR)

#### Women: special circumstances

Finally, women mentioned that some situations, such as a family member being sick, also influenced the way that they eat, especially when there is an NCD. For instance, one woman said

… I think that this can cause damage to my husband [to explain why she does not eat cakes]. (WJR)

In this same sense, women modified their diet if they had a chronic illness, although they admitted that they do not always follow their diets. A woman said:

… because I am chronic [in references that she has diabetes] … but I am stubborn, and one does not follow them [referring to recommendations]. (WAC)

Most HCPs mentioned that women experience anxiety due to stressful situations at home or at work, and use food as a means of escaping or gaining a sense of satisfaction. Most of the time, the women did not recognize that anxiety was affecting them and they also did not know the causes of their anxiety.

#### HCPs: they should value themselves

HCPs expressed the perception that most women have low self-esteem and this influences their beliefs about their ability to follow healthy diets. For example, one provider said:

I think that they [women] believe the merit is never due to them, it is for someone else, they maybe think that it is because the scale is broken … but never due to their effort …. (HCP IA)

#### HCPs: mental health influences

In this same sense, HCPs stated that the mental health of a woman can affect her ability to follow dietary recommendations. For instance, one HCP shared:

If you look at their diagnoses, always you are going to find a diagnosis associated with some psychological problem …. (HCP DG)

#### Government recommendations do not reach women's diet

Governmental programs and recommendations were also mentioned by the HCP in the sense that they fail to assist women to eat healthily. Sometimes the resources to develop these programs are scarce and there are few related specifically to the eating behaviors of women and their family. According to HCPs, government intervention programs have achieved limited success in certain segments of the population but not in others. Furthermore, attendance at these programs is often low, despite the fact that they are offered free of charge. Many HCPs in this study believed that both government guidelines and approved intervention programs need to be improved.

## Discussion

This study explored the perception of women and HCPs about the determinants affecting the eating behaviors of these women. Areas of significant agreement between women and HCPs were found. In order to achieve a degree of agreement between HCPs and women, HCPs need to have good communication skills, should be able to listen to the women, and be able to understand the most important determinants of eating behaviors. Similarly, women should be free to express their opinions, worries, or thoughts to their HCPs. Coran, Koropeckyj-Cox, and Arnold (2013) indicated that such concordance is dependent on the degree of the relationship between physicians and each person they care for. Consistent with this premise, in this study for many of the determinants we found a degree of agreement of consensus between women and their HCPs. This suggests that effective communication exists between them and that a person-centered communication model is being successfully implemented. Key factors in achieving this concordance include the individual care model adopted by the health care center in which a multidisciplinary team of HCPs coordinates interactions with the population. With this model, multiple encounters with them and other family members allow for a better knowledge of the people under their care (Ministerio de Salud de Chile, 2012). The primary health care system has been shown to be an ideal setting for the facilitation of this type of effective person-centered communication (Helitzer et al., 2011).

Among the consensus factors identified by both groups in this study, the role of the family was considered to be especially important. This is despite the fact that many women did not immediately appreciate its influence as a determinant of their eating behaviors. For women, the influence of family was only observed by indirect references to family members that emerged in the interviews. However for HCPs, the influence of family was more overt. It is important that the HCPs take advantage of their strong rapport with these women in order to help them realize how these factors may be affecting their eating behaviors and how women need to take into consideration family concerns when making decisions if they are to successfully change eating behaviors. Although this study only explored women's roles as women, and not specifically their roles as mothers or wives, it was difficult to separate these confounding factors from one another. It is likely that multiple roles exert important influences on these women. For HCPs, to understand eating determinants in women required that they also understand the eating behaviors of other members of the family.

Among the topics HCP and women disagreed on, habits and traditions were mentioned only by the women. As such, the HCPs could explore strategies to change unhealthy habits or traditions or to modify them in such a way as to minimize their adverse health effects. It is important for women to recognize how their anxiety and low self-esteem may need to be addressed in order to improve their overall health status. The fact that some factors are mentioned by one group and not the other may indicate that there are some factors that are more difficult for the women to express to the HCPs or, alternatively, for the HCPs to understand.

It is important that government evidence-based recommendations regarding food and eating behavior reach the target population. In the interviews, very few women mentioned being aware of public health recommendations related to following a healthy diet. An important role played by HCPs is that of communicating government guidelines to the women under their care. Our data suggest that this does not appear to be happening and that additional research is needed to help HCPs to communicate with their patients

This study underscores how effective communication between HCPs and their patients can be associated with better health outcomes. Studies show that when there is consensus between people and physicians regarding determinants, clinical outcomes have been shown to be more positive (Coran et al., 2013; Street, 2013). These improved health outcomes may result in improved psychosocial adjustment, and better adherence to treatment (Ishikawa et al., 2013; Venetis, Robinson, Turkiewicz, & Allen, 2009). In addition, studies have shown that when there is good communication, people tend to remember and adhere to recommendations given by their physician (Kaplan, Greenfield, Gandek, Rogers, & Ware, 1996; Ong, DeHaes, Hoos, & Lammes, 1995). For example, a study among individuals with diabetes found that a positive perception of the relationship between people and HCPs was associated with a better adherence to healthy diet and exercise plans (Maddigan, Majumdar, & Johnson, 2005). Good communication is associated with higher patient activation and participation during the encounter and treatment (Alexander et al., 2012) including overall individuals satisfaction (Oliveira et al., 2012).

HCPs continue to be the most important source of information about health and wellness. People prefer to discuss these issues with HCPs rather than other people (Friedman, Thomas, Owens, & Hébert, 2012). Studying the communication that occurs between HCPs and the people they care for can help to explain why some people are able to change their behaviors, and why others are not (Ciechanowski & Katon, 2001).

The findings from this study may guide HCPs regarding communication with their patients, how they understand certain behaviors, and their contribution to changing unhealthy eating patterns. In addition, they can reflect on the individual challenges and barriers found to be most problematic for specific patients and to help them to develop a strategy for overcoming these obstacles. Behaviors are complex because they are not influenced by only one factor, but are determined by multiple internal and external influences. Therefore, HCPs require the communication skills needed to obtain this information from their patients and take these factors into account when working to jointly develop an effective behavior change strategy. Understanding these processes will allow HCPs and health educators to create more focused and efficient interventions to improve the health of their communities.

This study extends public health research in several ways. Few studies have adopted qualitative designs to research the nature of communication between patients and HCPs related to eating behaviors. In addition, no other studies have used the photo-elicitation technique to assist people in telling their stories. Future studies could continue to use a combination of qualitative and quantitative approaches to further explore the degree of concordance between HCP and their patients. One potential limitation of this study was that the sample of women and HCPs was small and was obtained from only one health care center, making it difficult to generalize these findings to other centers in Chile. Furthermore, most HCPs interviewed were women, which could impact their ability to empathize with the women.

## References

[CIT0001] Alarcón A, Torres A, Barna R (2012). La atención primaria en salud, nuevos enfoques y perspectivas. Hallazgos desde un programa de formación y capacitación [The primary health care, new approaches and perspectives. Findings from a education and training program]. Revista Iberoamericana de Estudios Municipales.

[CIT0002] Alexander J. A, Hearld L. R, Mittler J. N, Harvey J (2012). Patient-physician role relationships and patient activation among individuals with chronic illness. Health Services Research.

[CIT0003] Anderson E, Winett R, Wojcik J (2007). Self-regulation, self-efficacy, outcome expectations, and social support: Social cognitive theory and nutrition behavior. Annals of Behavioral Medicine.

[CIT0004] Baker T. A, Wang C. C (2006). Photovoice: Use of a participatory action research method to explore the chronic pain experience in older adults. Qualitative Health Research.

[CIT0005] Barberia A. M, Attree M, Todd C (2008). Understanding eating behaviours in Spanish women enrolled in a weight-loss treatment. Journal of Clinical Nursing.

[CIT0006] Bergsten U, Bergman S, Fridlund B, Arvidsson B (2011). “Delivering knowledge and advice”: Healthcare providers’ experiences of their interaction with patients’ management of rheumatoid arthritis. International Journal of Qualitative Studies on Health and Well-Being.

[CIT0007] Bhuyan S (2011). Do consumers’ attitudes and preferences determine their FAFH behavior? An application of the theory of planned behavior. Agribusiness.

[CIT0008] Bisogni C, Jastran M, Seligson M, Thompson A (2012). How people interpret healthy eating: Contributions of qualitative research. Journal of Nutrition Education and Behavior.

[CIT0009] Braun V, Clarke V (2006). Using thematic analysis in psychology. Qualitative Research in Psychology.

[CIT0010] Cegala D. J, Street R. L, Clinch C. R (2007). The impact of patient participation on physicians’ information provision during a primary care medical interview. Health Communication.

[CIT0011] Ciechanowski P. S, Katon W. J (2001). The patient-provider relationship: Attachment theory and adherence to treatment in diabetes. American Journal of Psychiatry.

[CIT0012] Coran J. J, Koropeckyj-Cox T, Arnold C. L (2013). Are physicians and patients in agreement? Exploring dyadic concordance. Health Education & Behavior.

[CIT0013] Dresler-Hawke E, Veer E (2006). Making healthy eating messages more effective: Combining integrated marketing communication with the behaviour ecological model. International Journal of Consumer Studies.

[CIT0014] Fernstrom M. H, Reed K. A, Rahavi E. B, Dooher C. C (2012). Communication strategies to help reduce the prevalence of non-communicable diseases: Proceedings from the inaugural IFIC Foundation Global Diet and Physical Activity Communications Summit. Nutrition Reviews.

[CIT0015] Friedman D. B, Thomas T. L, Owens O. L, Hébert J. R (2012). It takes two to talk about prostate cancer: A qualitative assessment of African American men's and women's cancer communication practices and recommendations. American Journal of Men's Health.

[CIT0016] Glanz K, Rimer B, Viswanath K (2008). Health behavior and health education: Theory, research, and practice.

[CIT0017] Harper D (2002). Talking about pictures: A case for photo elicitation. Visual Studies.

[CIT0018] Harrington J (2004). Improving patients’ communication with doctors: A systematic review of intervention studies. Patient Education and Counseling.

[CIT0019] Helitzer D. L, Lanoue M, Wilson B, De Hernandez B. U, Warner T, Roter D (2011). A randomized controlled trial of communication training with primary care providers to improve patient-centeredness and health risk communication. Patient Education and Counseling.

[CIT0020] Holtrop J. S, Dosh S. A, Torres T, Thum Y. M (2008). The community health educator referral liaison (CHERL): A primary care practice role for promoting healthy behaviors. American Journal of Preventive Medicine.

[CIT0021] Instituto Nacional de Estadistica (2013). VII Encuesta de presupuesto Familiar [VII Survey of family budget].

[CIT0022] Ishikawa H, Hashimoto H, Kiuchi T (2013). The evolving concept of “patient-centeredness” in patient-physician communication research. Social Science & Medicine.

[CIT0023] Kaplan S, Greenfield S, Gandek B, Rogers W, Ware J. E (1996). Characteristics of physicians with participatory decision-making styles. Annals of Internal Medicine.

[CIT0024] Kothe E. J, Mullan B. A, Butow P (2012). Promoting fruit and vegetable consumption. Testing an intervention based on the theory of planned behaviour. Appetite.

[CIT0025] Lindsay A. C, Sussner K. M, Greaney M. L, Peterson K. E (2009). Influence of social context on eating, physical activity, and sedentary behaviors of Latina mothers and their preschool-age children. Health Education & Behavior.

[CIT0026] Ma Y, Olendzki B, Pagoto S, Hurley T, Magner R, Ockene I (2009). Number of 24-hour diet recalls needed to estimate energy intake. Annals of Epidemiology.

[CIT0027] Maddigan S. L, Majumdar S. R, Johnson J. A (2005). Understanding the complex associations between patient–provider relationships, self-care behaviours, and health-related quality of life in type 2 diabetes: A structural equation modeling approach. Quality of Life Research.

[CIT0028] Ministerio de Salud de Chile (2010). Encuesta Nacional de Salud 2009–2010 [Chilean National Health Survey 2009–2010].

[CIT0029] Ministerio de Salud de Chile (2012). Orientaciones para la implementacion del modelo de atencion integral de salud familiar y comunitaria [Guidelines for the implementation of the comprehensive health care model in family and community].

[CIT0030] Nishida C, Uauy R, Kumanyika S, Shetty P (2004). The Joint WHO/FAO Expert Consultation on diet, nutrition and the prevention of chronic diseases: Process, product and policy implications. Public Health Nutrition.

[CIT0031] Olivares C. S, Bustos Z. N, Moreno H. X, Lera M. L, Cortez F. S (2006). Actitudes y practicas sobre alimentacion y actividad fisica en ninos obesos y sus madres en Santiago, Chile [Food and physical activity attitudes and practices in obese children and their mother in Santiago, Chile]. Revista Chilena de Nutrición.

[CIT0032] Oliveira V. C, Refshauge K. M, Ferreira M. L, Pinto R. Z, Beckenkamp P. R, Negrao Filho R. F (2012). Communication that values patient autonomy is associated with satisfaction with care: A systematic review. Journal of Physiotherapy.

[CIT0033] Ong L. M, DeHaes J. C, Hoos A. M, Lammes F. B (1995). Doctor-patient communication: A review of the literature. Social Science & Medicine.

[CIT0034] Orji R, Mandryk R. L (2014). Developing culturally relevant design guidelines for encouraging healthy eating behavior. International Journal of Human-Computer Studies.

[CIT0035] Patton M. Q (2002). Qualitative research and evaluation methods.

[CIT0036] QSR International (2014). NVivo 10 research software for analysis and insight. http://www.qsrinternational.com.

[CIT0037] Shumaker S, Ockene J, Riekert K (2009). The handbook of health behavior change.

[CIT0038] Street R. L (2013). How clinician-patient communication contributes to health improvement: Modeling pathways from talk to outcome. Patient Education and Counseling.

[CIT0039] Swift J. A, Tischler V (2010). Qualitative research in nutrition and dietetics: Getting started. Journal of Human Nutrition and Dietetics.

[CIT0040] Troncoso P. C, Amaya P. J. P (2009). Factores sociales en las conductas alimentarias de estudiantes universitarios [Social factors in feeding behaviors of university students]. Revista Chilena de Nutrición.

[CIT0041] Venetis M. K, Robinson J. D, Turkiewicz K. L, Allen M (2009). An evidence base for patient-centered cancer care: A meta-analysis of studies of observed communication between cancer specialists and their patients. Patient Education and Counseling.

[CIT0042] Wanzer M, Booth-Butterfield M, Gruber K (2004). Perceptions of health care providers’ communication: Relationships between patient-centered communication and satisfaction. Health Communication.

[CIT0043] World Health Organization (1978). International Conference on Primary Health Care, Alma-Ata, USSR. Declaration of Alma-Ata.

[CIT0044] World Health Organization (2005). Preparing a health care workforce for the 21st century: The challenge of chronic conditions.

[CIT0045] World Health Organization (2014). Unhealthy diet. http://www.who.int/gho/ncd/risk_factors/unhealthy_diet_text/en/.

[CIT0046] Yamasaki J (2010). Picturing late life in focus. Health Communication.

[CIT0047] Zhao X, Villagran M. M, Kreps G. L, McHorney C (2012). Gain versus loss framing in adherence-promoting communication targeting patients with chronic diseases: The moderating effect of individual time perspective. Health Communication.

